# Memory loss in Alzheimer's disease: are the alterations in the UPR network involved in the cognitive impairment?

**DOI:** 10.3389/fnagi.2014.00008

**Published:** 2014-01-30

**Authors:** Claudia Duran-Aniotz, Gabriela Martínez, Claudio Hetz

**Affiliations:** ^1^Faculty of Medicine, Biomedical Neuroscience Institute, University of ChileSantiago, Chile; ^2^Program of Cellular and Molecular Biology, Center for Molecular Studies of the Cell, Institute of Biomedical Sciences, University of ChileSantiago, Chile; ^3^Neurounion Biomedical FoundationSantiago, Chile; ^4^Department of Immunology and Infectious diseases, Harvard School of Public HealthBoston, MA, USA

**Keywords:** Alzheimer's disease, UPR signaling pathways, memory, ER stress, memory impairment

Alzheimer's disease (AD) is a progressive and devastating age-related neurodegenerative disorder, involving memory loss and the extracellular deposition in the brain of misfolded and aggregated amyloid beta (Aβ) peptide (Holtzman et al., 2011). The molecular mechanism that triggers AD is not completely understood. The AD neuropathological process begins many years before the clinical onset with general alterations in protein homeostasis (referred to as proteostasis) among other effects. Recent evidence suggests that disturbances in the normal function of the secretory pathway and the occurrence of endoplasmic reticulum (ER) stress may represent a common pathological feature of familial and sporadic AD (Cornejo and Hetz, 2013). ER stress engages an adaptive reaction known as the unfolded protein response (UPR) which modulates many aspects of ER proteostasis to decrease the unfolded protein load (Walter and Ron, 2011). Under conditions of irreversible or chronic ER stress the UPR shifts its signaling toward induction of apoptosis.

Aβ oligomers are known to induce neuronal loss and dysfunction (Mucke and Selkoe, 2012) and impair synaptic plasticity and memory in animal models of AD (Cleary et al., 2005; Shankar et al., 2008). In this line, whether ER stress causes cognitive impairment remained poorly studied until very recently. Besides, interesting novel concepts are emerging where ER stress may actually operates upstream of the generation of Aβ as part of the etiology of the disease (Yoon et al., 2012). Could these findings provide insights about new points for disease intervention? Many recent studies have developed small molecules and gene therapy strategies to alleviate ER stress *in vivo*, which offers interesting future applications for the development of clinical trials in AD and other diseases (Hetz et al., 2013).

Medial temporal lobe areas, such as the hippocampus and entorhinal cortex, are the first regions affected during the progression of AD, contributing to the occurrence of dementia in affected patients. Under diverse stress conditions, including ER stress, inhibition of protein synthesis operates as a survival pathway that is mediated by the phosphorylation of eukaryotic translation initiator factor 2α (eIF2α), referred to as the “integrated stress response.” Of note, the process of memory consolidation and synaptic plasticity involve active protein synthesis, among other events (Costa-Mattioli et al., 2009). In fact, several studies have shown that exacerbated phosphorylation of eIF2α induces cognitive impairment (Costa-Mattioli et al., 2005, 2009; Jiang et al., 2010). In agreement with this findings, an elegant recent study demonstrated that decreasing the expression of two of the eIF2α kinases, double-stranded RNA-activated protein kinase (PKR)-like endoplasmic reticulum kinase (PERK) and General control non-derepressible-2 (GCN2), improve cognitive function and synaptic plasticity in an AD transgenic mouse model (Ma et al., 2013). In addition, targeting another eIF2α kinase termed dsRNA-dependent protein kinase (PKR), can also improve learning and memory processes at basal levels (Zhu et al., 2011), similarly to GCN2 deficient animals. Consistent with these finding, another recent report demonstrated that brain inflammation in AD models engages PKR to induce synaptic loss and memory impairment (Lourenco et al., 2013). In that study the authors also showed that Aβ oligomers alters insulin signaling leading to memory deficits through a mechanism involving the proinflammatory cytokine tumor necrosis factor (TNF)-α. Of note, PERK deficiency in the nervous system did not alter learning and memory-related processes at basal levels, and only impacted cognition in the context of AD models when ER proteostasis is altered (Ma et al., 2013). Importantly, these results solved an important question since they indicated that despite of reducing the adaptive activity of one branch of the UPR on a model of AD, this genetic manipulation improved cognitive aspects of AD without affecting the ability of cells to survive under the stress conditions generated by the accumulation of amyloid beta. Is the phosphorylation of eIF2α a key converging event involved in neuropathology and cognitive impairment in AD? Is this the molecular link between protein misfolding and neuroinflammation? These reports suggest the concept that modulation of protein synthesis through the eIF2α axis is directly involved in memory formation and could be also exploited as a target to reduce synaptic dysfunction in AD. Advances in this line were provided by a recent study identifying a small molecule called ISRIB that efficiently reduces the consequences of eIF2α phosphorylation and improve learning and memory in wild-type rats (Sidrauski et al., 2013). This potent inhibitor showed promising pharmacokinetic properties, it crossed the blood-brain barrier with no overall adverse effects to the animal. These findings raise the possibility that compounds that inhibit PERK signaling may offer interesting future applications for the development of clinical trials in AD. Fine-tuning the concentrations the compounds will be a challenging issue due to the dual impact of this signaling pathway on cell fate. In this line, PERK inhibitors have been recently shown to revert synaptic dysfunction and neurodegeneration in models of Prion disease (Moreno et al., 2013).

The most conserved signaling pathways of the UPR network is initiated by the ER stress sensor IRE1α. Active IRE1α splices the mRNA encoding the transcription factor X-box binding protein 1, shifting its coding reading frame that induces an active transcription factor termed XBP1s (Hetz, 2012). Last year, a polymorphism in the XBP1 promoter was described as a risk factor to develop AD. Remarkably, a global study to screen the universe of XBP1s-target genes revealed that this factor regulates a cluster of AD-related genes involved in the control of APP trafficking and processing (Acosta-Alvear et al., 2007). Together, these studies suggest that a second UPR signaling branch may also contribute to AD through a different mechanism. Although it was shown that cortical brain areas from post-mortem tissue showed a significant increase in the splicing of XBP1 mRNA (Lee et al., 2010), a recent report showed that XBP1 mRNA did not reach levels of healthy age-matched controls, suggesting down-regulation of this factor in AD brains (Reinhardt et al., 2013). In terms of functional studies, a neuro-protective activity of XBP1 was proposed on two fly models of AD involving the expression Aβ or Tau (Loewen and Feany, 2010; Casas-Tinto et al., 2011). Ectopic expression of XBP1s suppressed Aβ neurotoxicity in flies, possibly by modulating calcium homeostasis.

ER stress in AD also engages another stress pathways through IRE1α governed by cJun N-terminal kinases (JNK). JNK is activated in neurons of AD post-mortem brain tissue, and a recent report proposed that the occurrence of ER stress in AD mouse models may positive feedback to enhance Aβ formation and amyloid deposition through activation of JNK (Yoon et al., 2012). This study opens the question of whether ER stress signaling may contributes to diverse aspects of the disease: APP metabolism, Aβ aggregation, neurodegeneration and cognitive impairment. These observations are interesting because they contrast with the results obtained after manipulation of the UPR in other disease models, where the pathway has protective effects against protein aggregation (Matus et al., 2011; Roussel et al., 2013). These findings highlight the need to systematically investigate the actual contribution of XBP1, IRE1, and other UPR components such as ATF6 to AD to further validate and define the exact contribution of this homeostatic pathway to the disease process. Still, the cause of abnormal ER stress in AD remains to be determined.

**Figure 1:**
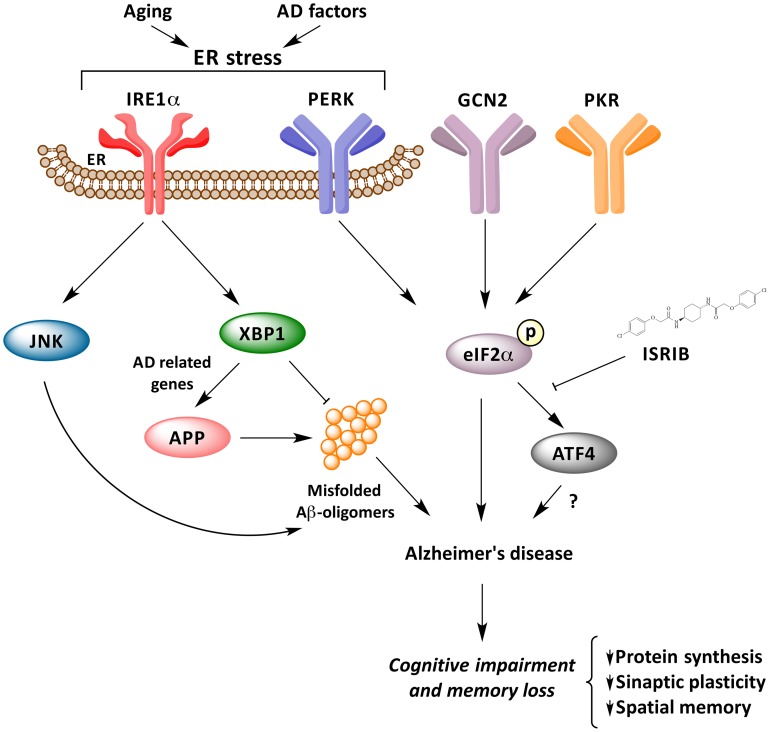
**UPR response underling memory consolidation in Alzheimer's disease**. Activation of ER stress signaling by abnormal protein misfolding activates several stress kinases leading to phosphorylation of eIF2α, inhibiting protein synthesis. Phosphorylation of eIF2α impairs synaptic function and cognitive processes. The IRE1α/JKN pathway may feed forward to enhance amyloid deposition and AD process, whereas XBP1 has neuroprotective effects against Aβ toxicity, and controls the expression of a cluster of AD-related genes.

Many important questions are still open in this emerging and growing field: (i) Is the IRE1α network, IRE1α/XBP1 and/or IRE1α/JNK pathways, involved in the consolidation and formation of memory? (ii) Do the activation of these pathways play a functional role in cognitive decline in AD? and, (iii) How is the UPR network as a whole related to the progression and pathogenesis of AD, APP processing and Aβ oligomers generation? Is neuroinflammation also converging into the IRE1a UPR axis? How can we consolidate that PERK signaling may have a dual and opposing activity in AD? All of the available evidence points to the fact that ER disturbances and UPR activation may facilitate and amplify both memory loss and protein aggregation on a vicious cycle that may turn initial adaptive UPR responses into a pro-degenerative factor (Figure 1). A systematic analysis is required to assess the exact contribution of each UPR signaling branches to AD to then define optimal targets for disease intervention.
